# Living sustainability, or merely pretending? From explicit self-report measures to implicit cognition

**DOI:** 10.1007/s11625-018-0561-6

**Published:** 2018-04-26

**Authors:** Gerald Steiner, Bernhard Geissler, Günther Schreder, Lukas Zenk

**Affiliations:** 1 Department for Knowledge and Communication Management, Faculty of Business and Globalization, Danube University Krems, Dr.-Karl-Dorrek-Straße 30, 3500 Krems, Austria; 2 Faculty of Geosciences, Geoengineering and Mining, University of Resources TU Bergakademie Freiberg, Akademiestraße 6, 09599 Freiberg, Germany

**Keywords:** Implicit cognition, Implicit association test (IAT), Explicit and implicit attitudes, Greenwashing, Sustainability awareness, Sustainable development goals (SDGs)

## Abstract

Recent research supports that a person’s self-reported explicit attitude is not necessarily consistent with their implicit attitude. However, in sustainability research, implicit cognitive measures are still at their early stages, and consider primarily singular aspects of sustainability. Here, we pose that the degree of congruence of individuals’ implicit and explicit attitudes represents the foundation of any organization’s sustainability culture. Although many organizations assert that sustainable development represents an important dimension of their vision and strategy, in reality, sustainable development often translates simply into explicit self-presentation and reputation. Traditional methods such as surveys lack information on implicit measures and—since they collect data based solely on the explicit knowledge of the respondents, which may be biased by social desirability and impression management—can therefore not determine the degree of congruence between explicit and implicit attitudes. We implemented a browser-based application of the Implicit Association Test (IAT) regarding sustainability as a reaction time-based cognitive measure supported by an interactive and activating process that was completed by 114 executives. Additionally, a questionnaire-based survey was conducted among them to investigate their explicit attitudes. We calculated Pearson correlation coefficients and conducted repeated measures MANOVA and principle component analysis. Our data analysis demonstrated low congruence between explicit and implicit sustainability orientations (Pearson’s *r* ranging from − 0.10 to 0.31). Potential explanations for our findings relate to the effects of impression management and individuals’ lack of cognitive processing of their own sustainability orientation. In sum, exploring the potential incongruence between explicit and implicit sustainability orientations helps narrow an important knowledge gap and provides a basis for rethinking the impact of internal and external learning processes within and between organizational systems, society, and science.

## Explicit and implicit attitudes toward sustainable development: why disentangling the two thinking modes may be key in moving toward the 2030 agenda for sustainable development

The perception of stakeholders regarding sustainable development plays a crucial role particularly for attaining the sustainable development goals (SDGs), which replace and expand upon the United Nations’ previous road map, the Millennium development goals (MDGs) and which now not only focus on developing countries, but include the developed world as well.

Examples for the perception of issues related to sustainable development can be found in various domains of sustainability research such as in risk perception (Weber et al. 2001; Weber 2012) or in corporate sustainability assessment (Weber and Banks 2012) and sustainable entrepreneurship (e.g., Provasnek et al. 2016a, b). In our investigation, we analyzed the underlying explicit and implicit attitudes toward sustainable development, and the correlation between these two dimensions.

The implicit cognitive dimension describes processes that occur without ourselves being consciously aware of them or without consciously controlling them and that influence perception, judgment, and action (Nosek et al. 2011). Implicit measures assess mental constructs—attitudes, stereotypes, self-esteem, and self-concepts—distinct from explicit self-reporting; although the underlying constructs are related, implicit measures help clarify different aspects of behavior not accounted for by the corresponding explicit measures (Greenwald and Banaji 1995; Nosek et al. 2011). Regarding its application, there is evidence that implicit measures, compared to introspectively derived explicit measures, are the more appropriate choice for dealing with people’s limitations regarding their motivation and opportunity to report mental content (e.g., related to social desirability) as well as their ability to translate it into verbal/written form or their inability to access introspection because of a lack of awareness (Nosek et al. 2011; Wilson and Brekke 1994).

Successful sustainability strategies can be strongly determined by executives’ implicit as well as explicit attitudes toward sustainability. The implicit cognitive dimension is of particular interest since “most of human cognition occurs outside of conscious awareness or conscious control” (Nosek et al. 2011, p. 152). However, it may differ from their explicitly proclaimed positive value of sustainability. The implicit association test is commonly used in the context of, e.g., addiction, race, and stereotypes. In sustainability research, studies have tended to use implicit measures to focus on consumer attitudes and product preferences, such as food or cosmetic consumption, recycling behavior, and consumers’ attention regarding carbon footprint information (Beattie and Sale 2011; Geng et al. 2013; Messner and Vosgerau 2009; Prestwich et al. 2011). The main reason for focusing on consumer behavior is its high impact on the environment (Beckham and Voyer 2014). In contrast, research on managerial attitudes in the context of sustainability is sparse and focuses on very narrow topics such as investor decisions on renewable energy (Chassot 2015). However, the significance of implicit attitudes of decision-makers in organizations also deserves closer examination.

An organizational phenomenon related to the potential incongruence of executives’ sustainability attitudes is known as greenwashing, which—in the context of corporate social responsibility (CSR)—may also be considered as “symbolic” CSR in contrast to “substantive” and “authentic” CSR (Donia and Tetrault Sirsly 2016). The phenomenon of greenwashing has been described in the fields of CSR and sustainability research (Donia and Tetrault Sirsly 2016; Mahoney et al. 2013; Reilly and Hynan 2014) but without focusing on the synergetic interdependence, respectively, the incongruence of explicit and implicit sustainability orientations of the executive as an individual. In our view, greenwashing expresses an incongruence between the reputational intention and the actual, real sustainability performance of the company, which may be partially attributed to the incongruent explicit and implicit sustainability orientation of its executives. Based on this discussion, our core research question is whether people tend to associate sustainability with positive feelings on a subconscious level. In the “Method” section, we will outline why this research question requires indirect methods that extend beyond conscious self-reporting.

Sustainability is an extensive systemic phenomenon that includes different levels of society. We focus our study on the bottom-up process in which individuals at the organizational level are key in moving toward sustainable development, but we go one step further. Because of social desirability and impression management in our society, people may overestimate their positive attitudes toward sustainability. This is the reason why we analyze not only their explicit self-reporting but also their implicit affective associations with sustainability—to start on the deepest human level, that of unconscious and implicit thinking.

Although corporate reputation, according to the annual Global McKinsey Survey, is a driving force for addressing sustainability (Bonini 2012), it is not necessarily an indication for greenwashing. Companies increasingly integrate sustainability principles into their business practices that go far beyond the concern of reputation management, i.e., by pursuing goals to attain both process and product improvements and to contribute to the short- and long-term value of their organization. A possibility for companies to differentiate themselves from competitors is to assess and document their sustainability performance along the way. This calls for an evaluation of sustainability performance according to social, environmental, and economic objectives within a company, over time, or across and within industries (Cohen et al. 2008; Miles et al. 2009). To transfer sustainability-related visions and activities to company’s stakeholders, sustainability reporting remains an important channel that is increasingly recognized as a cornerstone of corporate sustainability. Sustainability reporting originated from financial-reporting complements to the standard-setting Global Reporting Initiative (GRI) (Hahn and Kühnen 2013). The “de facto global standard” for sustainability reporting (KPMG 2011) emerged in the late 1990s and is currently in its fourth generation (G4). The latest KPMG survey, for which 45 countries were surveyed, showed that 60% of all CSR reports reference the GRI guidelines. From the 250 largest companies based on annual revenue, almost three quarters (74%) used the GRI framework; the overall sustainability-reporting rate for these companies was 92%. While reporting was originally voluntary, nowadays the main driver is of a legislative nature, and there is a growing trend in regulations that require companies to publish non-financial information. A further trend, found especially among the world’s largest companies, is external independent assurance (63%) on the reports that are typically based on self-assessment (KPMG 2015).

It also indicates that an organization’s sustainability orientation is increasingly tied to the sustainability orientation of its executives. If executives’ sustainability orientations demonstrate incongruence between explicit and implicit attitudes, it could be indicative of their tolerance for, or even encouragement of, greenwashing as a possible strategic option in the organizational context. Here, the explicitly proclaimed positive sustainability orientation is juxtaposed with the executive’s implicit attitudes for sustainable development. The need to investigate both the explicit and implicit sustainability orientations of various executives and stakeholders beyond executives becomes evident at the organizational level as well. For example, (1) companies are overwhelmingly considered to be the cause of sustainability challenges rather than their drivers of possible solutions; on the other hand, (2) companies themselves identify opportunities for more eco-efficient strategic options or cost reductions within their production processes; furthermore, (3) stakeholders are acting as drivers for a stronger sustainability orientation of companies by calling for sustainable products and service innovations, including more sustainable technologies or the company’s engagement in popular sustainability initiatives (Scherer et al. 2013; Whiteman et al. 2013).

The need for a thorough understanding of explicit and implicit sustainability orientations becomes even more evident at the global level by considering the most recent sustainable development goals (SDGs). On September 25, 2015, under the guidance of the United Nations (UN), “countries adopted a set of goals to end poverty, protect the planet, and ensure prosperity for all as part of a new sustainable development agenda … to be achieved over the next 15 years” (United Nations 2015, p. 1). These new sustainable development goals (SDGs) replace and expand upon the United Nations’ previous road map, the millennium development goals (MDGs) adopted in 2000. Whereas the MDGs focused mainly on developing countries, the SDGs have the potential to have a global impact by including the developed world as well. The agenda itself comprises 17 key initiatives, e.g., to ‘‘strengthen the means of implementation and revitalize the Global Partnership for Sustainable Development” (United Nations 2015). To manage the complexity related to the multidimensionality of these 17 goals in conjunction with their diverse effects on heterogeneous stakeholder groups from different cultures, we suggest that a better understanding of explicit and implicit attitudes at the level of the individual is crucial for the cross-boundary collaborations needed to attain these goals over the next 15 years.

While initiatives like the SDGs address the global level of sustainability, specific initiatives on an organizational level can also be found, depending on factors such as size, location, sector, or type of ownership: The UN Global Compact (UNGC), for example, represents the world’s largest voluntary corporate sustainability initiative, with close to 8000 corporate participants in more than 140 countries. The two main objectives (“Mainstream the ten principles in business activities around the world” and “Catalyze actions in the support of broader UN goals, including the MDGs”) are based on the four main pillars of human rights, labor, the environment, and anti-corruption (United Nations 2014).

To enhance the probability of the success of the SDGs and UNGC, a top-down policy approach will not be sufficient to deal with societies’ heterogeneity and embedded complex challenges; instead, mutual learning processes across societal, cultural, and religious boundaries will be essential, as proposed by transdisciplinarity research (Scholz and Binder 2011; Scholz and Steiner 2015a, b, c) and other recent forms of co-creation that aim to utilize the intelligence and experience of all stakeholders involved (Provasnek et al. 2016b; Steiner 2008). Therefore, possibilities to attain knowledge about explicit and implicit sustainability orientations may be helpful for a “revitalized Global Partnership for Sustainable Development” as promoted by the UN, since this will enable the design of more appropriate communication and collaboration strategies within this heterogeneous global society.

The heterogeneous perceptions of societal stakeholders, organizations, and policy makers challenge the study of sustainability orientations. Furthermore, these diverging perceptions also raise questions about the comparative strength of implicit versus explicit cognitive orientations to better understand perceptions of pro-active sustainability orientations as well as their counterparts. For the development of sustainability policies at various levels of society, it is crucial to address a potential incongruence between explicit and implicit attitudes toward sustainability to foster a narrowing of “the gap between words and deeds” (Seele 2015, p. 1) through deeper knowledge about the individual’s conscious and unconscious attitudes toward sustainability.

## Explicit and implicit sustainability attitudes as a basis for collaborative learning for sustainable transitions

Transitioning towards sustainable development is complex and multidimensional (e.g., Martens and Rotmans 2002), and calls for mutual learning processes between science and society, which might be aided by applying, e.g., a transdisciplinary methodology that is based on key concepts of systems theory such as complexity, vulnerability, and resilience (Scholz and Marks 2001; Scholz and Steiner 2015b). The heterogeneity of systems in transition and their stakeholders requires organizational change processes and educational means for sustainability (Hoover and Harder 2015). These include new or adapted leadership concepts to acquire the needed acceptance for joint problem-solving processes (Steiner 2014), such as shared leadership (Lee et al. 2015) and co-leadership (Scholz and Steiner 2015a, b); more effective governance structures (Tukker and Butter 2007); and stakeholder engagement processes to promote sustainable innovation orientation (Ayuso et al. 2011) that can be supported by appropriate forms of internal and external communication (both face-to-face and virtual) as well as being used as an enabler for building trust among the stakeholders involved (Drucker 1992, 2006; Weber et al. 2009; Zapico-Goni 2007); and the associated consensus-building processes (Sharkey and Sharples 2001).

To enable the co-creation of an adequate design for such transition processes, a deeper understanding of explicit and implicit attitudes toward sustainability is a prerequisite, especially for executives. This becomes particularly obvious in the context of stakeholder engagement, collaborative problem solving, and consensus-building processes. For example, the better the knowledge about explicit and implicit attitudes, the more effectively consensus-building processes may be designed. However, thus far, sustainability science as a relatively new field is often limited to the assessment of explicit attitudes, and sustainability research is based mainly on questionnaire-based investigations, interviews, case-study research, and observations (Salas-Zapata et al. 2013; Spangenberg 2011).

## The assessment of implicit sustainability attitudes: a conceptual approach and state-of-the-art measures

Explicit and implicit measures predict different aspects of behavior. From a theoretical perspective, these different values can be caused by (1) distinct mental entities (dual-representation theories), suggesting distinct processing of explicit and implicit cognitive representations, such as attitudes (Strack and Deutsch 2004; Wilson et al. 2000) or (2) distinct types of measures (single-representation theories, e.g., Fazio and Olson 2003; Kruglanski and Thompson 1999). As the empirical validity of representation theories has not yet been resolved, Greenwald and Nosek (2008) treat explicit and implicit measures as two empirically distinct constructs (Greenwald et al. 2009, p. 28f). Karpinski and Hilton (2001) provided strong evidence that explicit measures (i.e., self-reporting) are different from implicit measures (i.e., the IAT), conducting three different studies to investigate theories of dual attitudes toward objects. Therefore, both measures should be taken into account to deepen the knowledge of complex cognitive processes that affect human decisions, and thereby, have further consequences for societal change.

Various implicit measures are commonly used in cognitive science, although the degree of usage, the range of fields relevant to their application, and their inclusion/exclusion boundaries show huge variations; a comprehensive review of measures for implicit cognition is provided by Nosek et al. (2011). The Implicit Association Test (IAT) (Banaji and Greenwald 2013; Greenwald and Farnham 2000; Greenwald et al. 1998), which is the core measure of implicit sustainability attitudes in our investigation, is a general purpose procedure for determining implicit attitudes by measuring the strength of automatic associations between different concepts. Hence, the IAT is a measurement for determining implicit attitudes and beliefs that may differ from explicitly expressed self-reporting because (1) people may be unwilling or (2) unable to report their attitudes (see above).

Compared to explicit self-reporting, the IAT measures automatically activated associations, and therefore, it is very difficult if not impossible for participants to feign, or fake, the results. To test these assumptions, various studies were conducted to examine, e.g., homosexual–heterosexual attitudes (Banse et al. 2001), shyness self-concepts (Asendorpf et al. 2002), or racial attitudes (Kim 2003). Participants in these studies were able to fake their self-reports but were not able to fake the IAT, even if they had been instructed to make a good impression in a job application scenario, e.g., to fake their anxiety level (Egloff and Schmukle 2002).

Since the first publication of the IAT in 1998, various studies have been conducted in regard to the psychometric properties of the IAT’s measures (Egloff and Schmukle 2002; Greenwald and Farnham 2000; Greenwald and Nosek 2001; Lane et al. 2007; Nosek et al. 2007; Rudman et al. 1999). Greenwald, Poehlman, Uhlmann, and Banaji (2009) conducted a meta-analysis to estimate the average predictive validity effect size of IAT measures regarding behavioral, judgement, and physiological measures, compared to self-reported measures. Based on 122 reports that included 184 independent samples, the effect size of the predictive validity of the IAT was, on average, 0.274, which is characterized as moderate. The predictive validity of explicit self-report measures was larger (0.361), but was reduced in the context of socially sensitive topics. Hence, although the implicit measures (IAT) overall had a lower effect size, they succeeded compared to the explicit measures when the questions involved socially sensitive topics (e.g., interracial behavior or intergroup behavior). Self-report measures for topics with a low score in social sensitivity had a high effect size (0.60), but for topics with a high score in social sensitivity, they had a low effect size (0.10). The IAT, in contrast, was robust in socially sensitive domains (Greenwald et al. 2009).

In recent years, the IAT has been used in various fields, e.g., discrimination in human resources, law enforcement, criminal justice, education or health care, and on a personal and system level. Especially in the latter case, in which large samples are used, IAT measures are appropriate to predict societally important topics, even with lower effect sizes (Greenwald et al. 2015). As an example, Nosek et al. (2002) analyzed the data from over 600,000 web-based IATs addressing stereotypical attitudes towards diverse social groups. Respondents had to classify black and white faces or names while at the same time classifying words of positive (e.g., “good”) or negative (e.g., “bad”) valence. Results showed the respondents’ automatic preference for one of these social groups. Similar tests on the preference of age were completed, as well as tests that measured the respondents’ implicit associations of male with science or career, and females with liberal arts or family. The tests were accompanied by questions on their attitudes toward the specific topic (e.g., “Please rate your attitude towards…”). The implicit (IAT) and explicit (question items) attitudes were positively correlated, though mostly at a rather low level (with Pearson’s *r* ranging from 0.08 to 0.52, on average 0.24), leading the authors to state that the differences between the two measurements “suggest a form of mental (and often unrecognized) dissociation between implicit and explicit feelings and thoughts.” (Nosek et al. 2002, p. 112).

Chassot et al. (2015) used the Implicit Association Test in the field of renewable energy sources to investigate the unconscious attitudes of investment behavior. They argue that unconscious attributes influence the decision-making of energy industry professionals, especially in a high-uncertainty investment context. Hence, they studied their explicit and implicit attitudes: in the IAT, the target categories “solar energy” and “natural gas” and the attributes “positive” and “negative” were used; for the explicit association measures, a 7-point Likert scale was used and participants indicated the extent to which they associate amongst other solar energy with “positive”. Correlating the implicit and explicit attributes with the investor’s behavior showed that the implicit variable had a stronger correlation with the investor behavior than the explicit variable. These results suggest that the use of an implicit measurement can provide a better understanding of investors’ decision-making than asking them explicitly.

In sustainability-related fields, positive relationships between implicit attitudes and decisions affecting health (Prestwich et al. 2011), brand choices (Messner and Vosgerau 2009), recycling behavior (Geng et al. 2013), and environmentally friendly food shopping were found (Beattie and Sale 2011). Beattie and McGuire (2015) studied how explicit and implicit attitudes predict the visual attention of consumers in regard to the carbon footprint information on product labels. They found no significant correlations between explicit attitudes and the eye fixation of participants on the footprint information. However, participants with a strong positive implicit attitude toward a low carbon footprint were more likely to fixate first on carbon footprint information. Similarly, Beattie and McGuire (2012) found a link between the implicit attitude toward the environment and unconscious patterns of eye movements. People with a strong positive implicit attitude toward low carbon products also spent more time looking at negative images of climate change. In comparison, explicit self-reporting did not predict the measured eye gaze. Most recently, Panzone, Hilton, Sale, and Cohen (2016) found that implicit attitudes toward sustainability seem to be activated in specific consumer choices and might be used to significantly predict consumer demands for bottled water.

Notably, most of the studies mentioned used implicit measures to focus on a specific area of sustainability, i.e., consumer consumption or recycling behavior. We therefore decided to study sustainability in this paper by taking a broader approach, i.e., integrating economic, ecological, social, institutional, and cultural sustainability.

## Method

### Study sample

To reach a sample of executives from different companies, we contacted 1085 persons, of which a total of 114 participants completed the IAT and the survey. We used two ways of recruitment: 827 participants of postgraduate courses of Danube University Krems were contacted via the internal e-learning platform Moodle or directly during a course. Of note, Danube University Krems is a University for Continuing Education with an exclusive focus on postgraduate education; its student body therefore almost exclusively comprises executives in managing positions, and the vast majority of all students have five or more years of work experience. Because of its focus on executives with work experience, students of Danube University Krems are on average 40 years old, work in various companies and attend specific courses as part of their extra-occupational postgraduate education (the University offers Master’s programs and certified programs only, but no Bachelor programs). In addition, to enhance the sample size, 258 executives from various companies in Austria were contacted by email through the Austrian Department of the Environment. The participants’ mean age was 39.9 years (SD = 10.1); 63 were females and 51 were males. On average, the participants had been employed for 8.6 years by their current company (SD = 7.4), and 84 participants reported holding an academic degree, while 20 reported having completed training in sustainability. For 34 participants, sustainability issues were at least part of the main responsibilities of their jobs; 37 reported that sustainability issues were part of their secondary responsibilities; and 43 stated that they did not have specific responsibilities in this area.

### Measures of attitudes

Whereas explicit attitudes can be analyzed based on questionnaires (either paper-based or digital), the measurement of implicit attitudes depends heavily on the availability of reliable automated procedures that can be performed outside the laboratory to keep them cost-efficient while allowing large-scale applications such as at companies or at organizational levels.

### Development and measures of explicit attitudes

The assessment of explicit sustainability attitudes in our study was conducted with an online questionnaire using LimeSurvey. The 41 questions were grouped into seven categories as follows: demographics of the participant, demographics of the company, attitude toward sustainability, sustainability key-performance indicators, sustainability certifications, digitalization, and continuing education. Part of these questions was aimed at the implementation of sustainability tools, certificates and indicators in organizations, and they used mainly an open-answer format. In this paper, we focus on the questions that were used to measure attitudes towards sustainable development and methods that promote sustainability as described in the following:

Study participants had to rate the importance of various sustainability aspects for themselves and for their respective organizations: “What significance has sustainability for you?” and “What significance has sustainability in your organization?” We distinguished between the following five categories: economical, ecological, social, institutional, and cultural sustainability. Similarly, we asked participants to rate their agreement with prescribing regulations and setting basic parameters to enforce or promote sustainable behavior in their respective organizations. Furthermore, all participants had to rate the importance of indicators, certificates, digitalization, and education for sustainability. All questions had to be answered on a 6-point Likert scale (from 0...very low to 5...very high) to quantify the attitudes of the participants.

Completed questionnaires were exported and processed using SPSS version 22.

### Development and measures of the sustainability IAT

For the current study, we focus on executives’ implicit attitudes toward sustainability. Although the domain of sustainability is not as socially sensitive as discrimination, a positive attitude toward a sustainable future is nevertheless socially desirable. As a consequence, we hypothesize a difference in explicit and implicit attitudes due to the effects of impression management. Because the degree of cognitive elaboration of the topic was found to have a moderating effect on the relationship between the IAT and explicit self-reports, we expect participants who are active or who have specific training in the field of sustainability to show higher correlations between the implicit measure and their self-reports.

The main research question for the study, namely, whether people tend to associate sustainability with positive feelings on an unconscious level, requires indirect methods that go beyond conscious self-reporting. The IAT is an appropriate tool to use to address this question, and we chose the most empirically tested version, the “Standard IAT”. For this test, it is necessary to use four simple categories that are distinguishable for fast decisions that are based on automatic or “fast thinking” processes (Kahneman 2012). For that reason, we used the categories “Sustainable” and “Unsustainable” as well as “Good” and “Bad”.

**Fig. 1:**
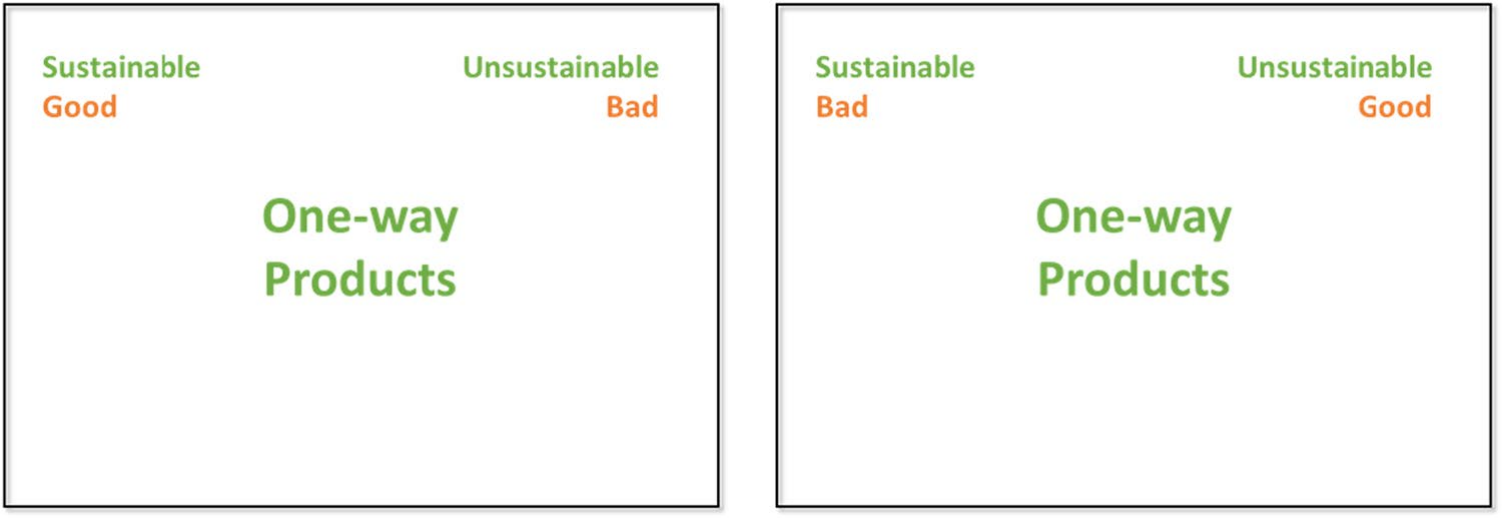
Sustainability IAT: test of congruent categories on the left and test of incongruent categories on the right. Stimuli are shown in the center

During the test, specific words (“stimuli”) are shown, and participants have to decide spontaneously to which base concept they fit: depending on the stimulus, they must thus choose between qualitative judgements (“Good” vs. “Bad”) or decide if the object in question matches the definition of “Sustainable” (see Fig. 1). Color-coding was used to indicate which category the stimulus belonged to. To generate suitable stimuli for the categories “Sustainable” and “Unsustainable,” we conducted qualitative interviews with multiple subject-matter experts to develop the IAT. Accordingly, we used a simple survey to identify which stimuli best fit the categories by asking different non-experts to rate how representative each of the words for each category was based on a Likert-type scale. For “Good” and “Bad,” we used stimuli suggested by Nosek, Banaji, and Greenwald (2002). In Table 1, we list all categories with the appropriate stimuli.

**Table 1:** Four categories with five stimuli per category for the Standard IAT

Sustainability IAT	
Sustainable	Unsustainable
Continuing education	Energy-intensive
Re-use	Short-term orientation
Economic sustainability	Speculation
Social commitment	Pure profit orientation
Product stewardship	One-way products

Good	Bad
Happy	Bad
Peace	Failure
Pleasure	Terrible
Joy	Pain
Laugh	Disgusting

The browser-based application of the Standard IAT comprises 7 phases (see Table 2). In phase 1, the categories “Good” and “Bad” are shown at the top of the monitor. In the center, the stimuli for “Good” or “Bad” (e.g., Happy, Terrible, etc.) are shown in randomized order. The participants are asked to hit “w” on the keyboard if the word matches the category on the left and to press “o” if the word matches the category on the right. In phase 2, the categories “Sustainable” and “Unsustainable” as well as the appropriate stimuli are shown. In phase 3, all four categories and stimuli are used, and the participants must decide spontaneously if a word fits on the left (“Sustainable” or “Good”) or on the right (“Unsustainable” or “Bad”) (see Fig. 1 on the left). Pressing the wrong key caused an error prompt on the screen and participants were instructed to press the correct key instead.

**Table 2:** The Standard IAT comprises 7 phases

Phase	Left category	Right category	No. of stimuli	Aim
Phase 1	Good	Bad	20	Preparation
Phase 2	Sustainable	Unsustainable	20	Preparation
Phase 3	Good	Bad	20	Preparation
	Sustainable	Unsustainable		
Phase 4	Good	Bad	40	Test
	Sustainable	Unsustainable		
Phase 5	Bad	Good	20	Preparation
Phase 6	Bad	Good	20	Preparation
	Sustainable	Unsustainable		
Phase 7	Bad	Good	40	Test
	Sustainable	Unsustainable		

The first three phases aim to prepare the participant to intuitively assign the words to the different categories. In phase 4, the reaction time for correct answers to 40 stimuli presented in random order is measured in milliseconds. In phases 5–7, the categories “Good” and “Bad” are transposed (see Fig. 1 on the right). After the preparation part in phases 5 and 6, the reaction time is measured again for 40 stimuli (see Table 2).

The underlying assumption of the IAT is that participants are able to respond more quickly to closely associated, congruent categories (phase 4) than to not closely associated, incongruent categories (phase 7). In our case, we hypothesized that “Sustainable and Good” as well as “Unsustainable and Bad” are associated as congruent categories by the participants (phase 4) and that “Sustainable and Bad” as well as “Unsustainable and Good” represent incongruent categories (phase 7) (see Panzone et al. 2016). Consequently, the average reaction time (rt) in phase 4 should be shorter than in phase 7, computed with a *D* value (the value of the different reaction time):

1 $$D\;{\text{value}}\;{\text{=}}\;\frac{{{\text{avg}}\left( {{\text{r}}{{\text{t}}_{{\text{incongruent}}}}} \right)\; - \;{\text{avg(r}}{{\text{t}}_{{\text{congruent}}}}{\text{)}}}}{{{\text{standard deviation(r}}{{\text{t}}_{{\text{incongruent}}}}\;{\text{+}}\;{\text{r}}{{\text{t}}_{{\text{congruent}}}}{\text{)}}}}$$

Incorrect answers as well as answers below a reaction time of 300 ms or above 10.000 ms were filtered (Beattie and McGuire 2015). To take into account order effects (such as practice and fatigue), the order of incongruent and congruent phases of the test was counterbalanced.

To collect data from various executives, a browser-based application of the IAT was required that was easy to use. Although different versions of software tools are available, we have not found a software program that is sufficiently designed to expect a high response rate (e.g., some software tools must be downloaded by the participants or require additional software to run the test). Because of this, we developed a browser-based tool in the course of an applied research project^1^ that allows participants to take the IAT directly using common web-browsers.

## Results

### Explicit data

**Fig. 2:**
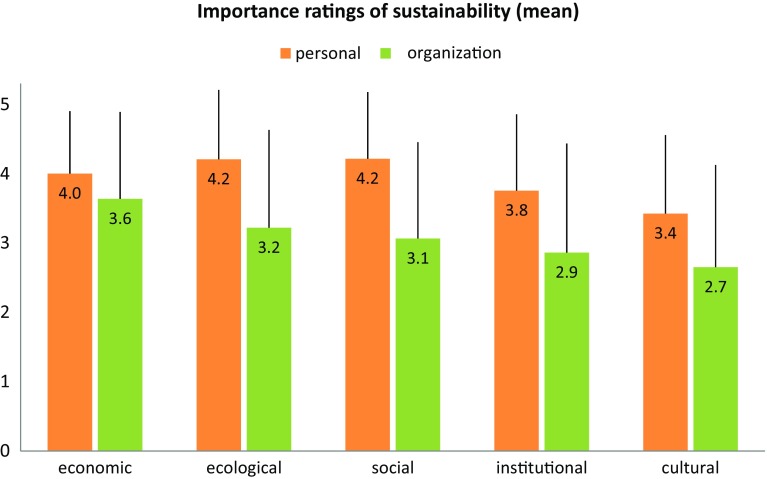
Subjective importance of sustainability—comparison of self-reports and assessment of own company (mean, standard deviation, 6-point Likert scale from 0...very low to 5...very high)

In a first step, we analyzed how the participants rated the importance of sustainability regarding its economic, ecological, social, institutional, and cultural domains. We compared these results to how participants rated the importance of these aspects of sustainability within their companies. The results are illustrated in Fig. 2 and highlight the generally high importance ratings.

A repeated measures MANOVA indicates that, in the view of most participants, their companies do not put as much emphasis on sustainability as they themselves do (*F*(5, 93) = 751.03; *p* < 0.01). Univariate comparisons reveal that this gap is manifested in all five sub-categories of sustainability and is strongest for social (*F* = 64.50, *p* < 0.01) and ecological sustainability (*F* = 45.83, *p* < 0.01) and weakest for economic sustainability (*F* = 5.47, *p* < 0.05). Participants’ levels of responsibility within their companies showed no effect on the ratings.

**Fig. 3:**
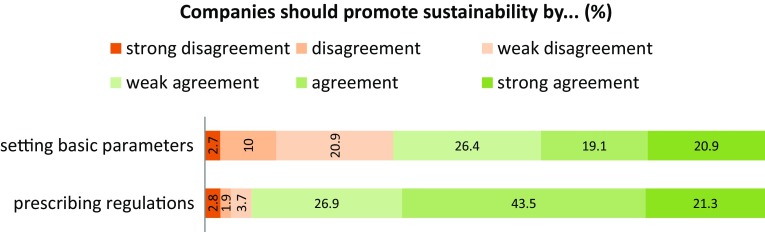
Approval for measures to promote sustainability

In more than 50% of all cases, the respondent´s company is perceived as only rarely discussing the topic of sustainability. This gap could explain the majority’s demand for companies to promote sustainability by setting parameters to support self-responsibility of employees as well as implementing regulations (see Fig. 3).

**Fig. 4:**
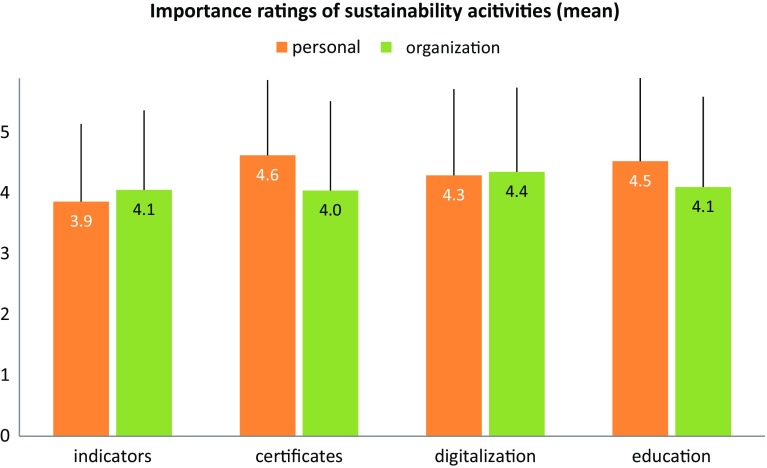
Subjective importance of different sustainability activities—comparison of self-reports and assessment of own company (mean, standard deviation, 6-point Likert scale from 0...very low to 5...very high)

Looking further into the details of how the participants value different organizational activities in the field of sustainability, the results show that certificates and education are rated highest (see Fig. 4). Interestingly, sustainability indicators are valued less than certificates are. Similar to the overall importance ratings, a significant difference between self-reports and assessment of the participants’ organizations can be observed (repeated measures MANOVA, *F*(4,88) = 6.36, *p* < 0.01). This gap apparently results from different ratings of certificates (*F* = 15.75, *p* < 0.01) and educational measures (*F* = 6.58, *p* < 0.05); differences concerning the other two categories (indicators and digitalization) were not significant.

### Implicit data (IAT) D

**Fig. 5:**
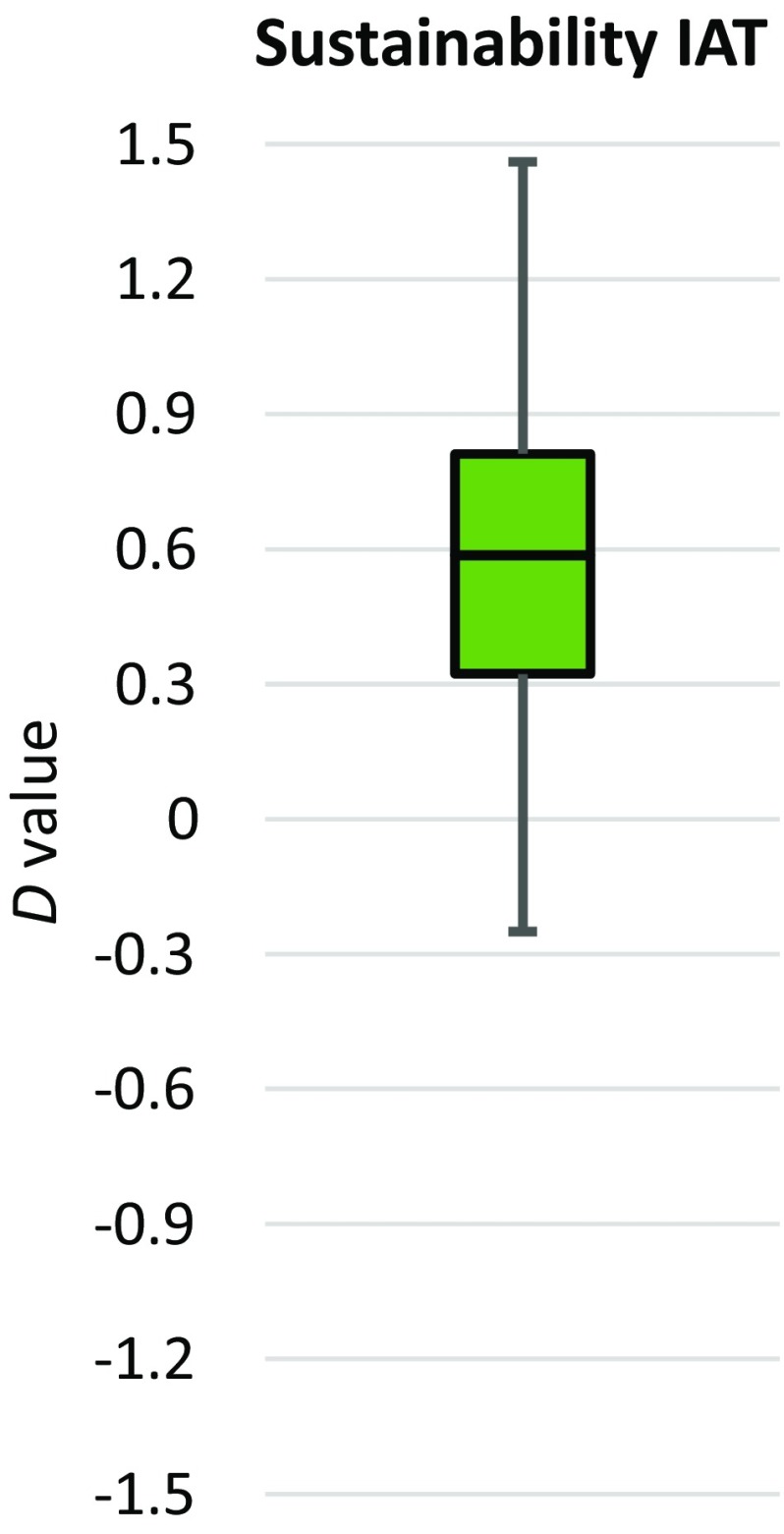
Box plot of IAT result (*D* value)

For the IAT, we calculated *D* values according to the procedure described by Greenwald et al. (2003). *D* values are standardized differences of the mean reaction times of the congruent and incongruent phases of the test (see Formula 1 in Sect. “Methods”). The differences between blocks 6 and 3 and blocks 7 and 4 were each divided by the pooled standard deviation, and both scores were averaged. In our design, high scores indicate a high associative strength between “Sustainable” and “Good”, and low scores indicate a high associative strength between “Sustainable” and “Bad”. The results demonstrate an average *D* value of 0.57 (SD = 0.32), ranging from − 0.18 to 1.46 (see Fig. 5). For comparison, very similar results were found by Panzone et al. (2016), with an average *D* value of 0.72 (SD = 0.29). As *D* values can be interpreted similar to Cohen’s *d* (Cohen 1988; Greenwald et al. 2003), we can use the IAT’s *D* value to estimate the strength of the effect—the result indicates a moderate-to-strong effect for the implicit association between sustainability and positive feelings.

At first glance, the data appear to match the general impression of positive attitudes toward sustainability. More precisely, we were interested in the relationships between explicit and implicit data as well as in the possible differences between them. Interestingly, virtually no significant correlations between *D* value and explicit self-reports were found (see Table 3).

**Table 3:** Correlation matrix (Pearson’s *r*)

	IAT *D* value	Economic sustainability	Ecological sustainability	Social sustainability	Institutional sustainability	Cultural sustainability	Sustainability addressed by company	Prescribing regulations	Setting basic parameters	Importance of indicators	Importance of certificates	Importance of digitalization	Importance of education
IAT *D* value	**1**	**0.17**	**0.00**	**− 0.03**	**0.11**	**0.05**	**− 0.02**	**0.04**	**− 0.08**	**0.15**	**− 0.10**	**0.03**	**− 0.05**
Economic sustainability		1	0.19*	0.15	0.24**	0.17	0.12	0.22*	0.09	0.18	0.10	0.07	0.16
Ecological sustainability			1	0.57**	0.31**	0.32**	0.30**	0.24*	0.18	0.12	0.24*	0.05	0.16
Social sustainability				1	0.41**	0.23*	0.08	0.22*	0.14	0.20*	0.26**	− 0.00	0.12
Institutional sustainability					1	0.57**	0.02	0.10	0.12	0.16	0.16	0.01	0.17
Cultural sustainability						1	0.11	0.24*	0.15	0.16	0.14	− 0.01	0.10
Sust. addressed by company							1	0.25**	0.31**	0.13	0.15	0.19*	0.17
Prescribing regulations								1	0.193*	0.207*	0.240*	0.154	0.07
Setting basic parameters									1	0.18	0.21*	0.16	0.11
Importance of indicators										1	0.45**	0.24*	0.38**
Importance of certificates											1	0.17	0.27**
Importance of digitalization												1	0.32**
Importance of education													1

*p < 0.05

**p < 0.01

**Fig. 6:**
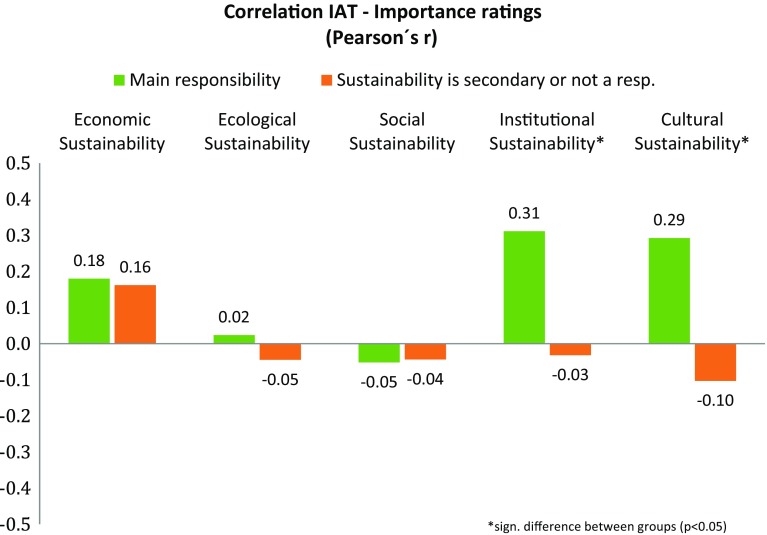
Correlation between IAT (implicit) and self-reporting (explicit) measures

To evaluate whether cognitive elaboration had any effect on the correlative relationship between the explicit and implicit measures, we performed a test for the comparison of correlation for independent groups based on Fisher’s *r*-to-*z* transformation (Eid et al. 2013). Participants who stated that sustainability was one of their main responsibilities were compared to participants who reported that sustainability was only a secondary responsibility or not a responsibility of theirs at all. Correlations between *D* values and the five dimensions of sustainability used in the questionnaire are depicted in Fig. 6. Possible effects of cognitive elaboration were found only for institutional (*z* = 1.66, *p* < 0.05) and cultural sustainability (*z* = 1.87, *p* < 0.05). A further test to analyze the influence of training was omitted due to the small number of participants who had actually received training in sustainability.

A principle component analysis (PCA) was performed and yielded good sampling adequacy (KMO = 0.75, *p* > 0.01). Seven main factors (see Table 4) could be extracted; cumulatively, they explain 71.85% of variance. Again, there is a pronounced difference between the perception of company values and participants’ own attitudes toward sustainability. While all data on the values represented by participants’ organization contribute to a single factor (with the sole exemption of digitalization), the individual attitudes are divided into four different factors as follows: opinion on sustainability measures, importance of institutional and cultural sustainability, desire for regulations, and importance of economic sustainability. The topics most-often associated with sustainability, i.e., ecological and social sustainability, are confounded with these factors. Data on the relevance of digitalization for questions pertaining to sustainability result in their own factor that combines personal attitudes and perceptions about participants’ employers. The IAT results represent a separate factor not correlated to any of the explicit data.

**Table 4:** Results of principle component analysis

Factor	Variance (%)	Items^a^
1. Assessment of company values	21.0	Sustainability addressed by company Ecological sustainability Social sustainability Institutional sustainability
2. Personal importance of sustainability measures	11.8	Certificates Measurements Training
3. Institutional and cultural sustainability	9.9	Institutional sustainability Cultural sustainability
4. Digitalization	9.5	Importance of digitalization (personal) Importance of digitalization (company)
5. Regulations	7.6	Prescribing regulations Setting basic parameters
6. Economic sustainability	6.4	Economic sustainability
7. Implicit attitude towards sustainability	5.6	IAT *D* value

^a^Based on factor loadings of 0.7 or more

Overall, the 114 executives who participated in this study (all from German speaking countries) showed rather positive explicit attitudes towards sustainability and related activities, i.e., at least more positive than they rated the corresponding attitudes within their own organizations. Implicit measures point in the same direction: a medium to strong positive implicit attitude towards sustainability was observed. However, correlations between explicit ratings and implicit measures were close to zero. The sole exception was economic sustainability (with a Pearson’s *r* of 0.18). Only in cases when respondents had a personal responsibility for sustainability issues, there were correlations with cultural and institutional sustainability. A PCA revealed several factors of sustainability-related attitudes, but the IAT *D* value was an isolated factor among those, further hinting at a dissociation between explicit and implicit attitudes.

## Discussion and outlook

In the introduction, we delineated that the sustainability orientation of executives is a powerful driver for sustainable development goals. On one hand, executives could promote the realization of reporting and other sustainability-related actions and play an important role in furthering their companies’ sustainability agenda. On the other hand, they could show a lack of awareness towards sustainability-related organizational challenges or even encourage greenwashing as an organizational strategy. We argue that a deeper understanding of the explicit and implicit attitudes of executives provides invaluable information for stakeholder engagement, collaborative problem solving, and consensus-building processes.

The observed very low correlation between the implicit association test (IAT) on sustainability and the survey data could stem from self-reporting bias and social desirability, but self-presentation is only one possible factor that influences the relationship between explicit and implicit measurements. Inaccurate explicit self-reports can also be the result of a lack of awareness of personal attitudes. Low motivation or few opportunities to engage in sustainability issues could lead to poor introspection and a limited cognitive elaboration of the topic. A third explanation, which cannot be fully discounted, is the homogeneity of the population and the associated lack of variance (Brunel et al. 2004). Because the IAT provides a better predictive quality when socially sensitive topics are involved (Greenwald et al. 2009), a meaningful next step could be to identify which aspects of sustainable behavior can be predicted with explicit reports and in which cases and contexts implicit measures such as the IAT provide a better predictive validity.

Regarding the latter, the comparison of implicit attitudes might relate also to economic approaches such as conjoint analysis, hedonic pricing, or contingent choice methods as potential preference methods of valuation. The IAT could help reveal underlying attitudes, which might explain observed choices, the willingness to pay for certain goods, or other outcomes resulting from valuation methods. Furthermore, some studies combined IAT results with the assignment of dollar values, analogous to contingent choice methods. In one example, Rahnev et al. (2007) and Caruso et al. (2009) used conjoint analyses to investigate consequences of stereotypes. These results showed that participants, although they explicitly stated having no preference regarding the gender of their supervisor, preferred male supervisors even if this meant a lower-pay job.

Nosek et al. (2007) point out that there is no evidence that the IAT “might serve as a lie detector, revealing associations that are more ‘real,’ ‘true,’ or accurate than self-report” (p. 282). Instead, results obtained by implicit measures can differ from explicit self-reporting because participants might not want to report their unconscious preferences or may not be aware of their implicit associations (i.e., without such a test procedure, one cannot be conscious about an unconscious association). Instead, implicit measures (i.e., the results from an IAT) are a different type of measure than explicit measures (Karpinski and Hilton 2001), and therefore, both should be considered as equally valuable methods that contribute to a more thorough understanding. Such an understanding can help to enhance collaboration efforts and co-creation processes in general (including transdisciplinary approaches) and specifically at the company level as well as at the international level (e.g., in association with collaborative initiatives related to the 17 initiatives of the SDGs). On the other hand, a more holistic comprehension of attitudes could also be misused, particularly when a browser-based applications for data collection is applied to realize one-sided interests (e.g., when the knowledge of specific attitudes as such is used against people participating in experiments).

This paper and the underlying experimental investigation have provided evidence that, to narrow “the gap between words and deeds” (Seele 2015, p. 1), a potential incongruence and/or the synergetic interplay between explicit and implicit attitudes toward sustainability need to be addressed. With this extended knowledge base, policies and specific interventions toward sustainability targets can be tailored and empirically evaluated more effectively and efficiently; these are valid for individuals at the organizational level (e.g., when implementing a company’s sustainability agenda) as well as at the international policy level (e.g., when implementing SDGs). Although the underlying investigation was conducted within a scientific setting, an application as a supportive “sustainability software tool” in organizational settings is feasible and promising.

## Footnotes

1
http://www.donau-uni.ac.at/spoc
